# Polyphenols from Brown Seaweeds (Ochrophyta, Phaeophyceae): Phlorotannins in the Pursuit of Natural Alternatives to Tackle Neurodegeneration

**DOI:** 10.3390/md18120654

**Published:** 2020-12-18

**Authors:** Mariana Barbosa, Patrícia Valentão, Paula B. Andrade

**Affiliations:** REQUIMTE/LAQV, Laboratório de Farmacognosia, Departamento de Química, Faculdade de Farmácia, Universidade do Porto, Rua de Jorge Viterbo Ferreira n.º 228, 4050-313 Porto, Portugal; mariana.nunes.barbosa@gmail.com (M.B.); valentao@ff.up.pt (P.V.)

**Keywords:** phlorotannins, multitarget, neuroprotection, neuroinflammation, Aβ amyloid, oxidative stress

## Abstract

Globally, the burden of neurodegenerative disorders continues to rise, and their multifactorial etiology has been regarded as among the most challenging medical issues. Bioprospecting for seaweed-derived multimodal acting products has earned increasing attention in the fight against neurodegenerative conditions. Phlorotannins (phloroglucinol-based polyphenols exclusively produced by brown seaweeds) are amongst the most promising nature-sourced compounds in terms of functionality, and though research on their neuroprotective properties is still in its infancy, phlorotannins have been found to modulate intricate events within the neuronal network. This review comprehensively covers the available literature on the neuroprotective potential of both isolated phlorotannins and phlorotannin-rich extracts/fractions, highlighting the main key findings and pointing to some potential directions for neuro research ramp-up processes on these marine-derived products.

## 1. Introduction

Despite the Sustainable Development Goals aiming to reduce premature mortality from non-communicable diseases by 2030, as the average life expectancy continues to rise, the prevalence of non-communicable neurological disorders is likely to increase. Neurological disorders are indeed one of the world’s largest causes of disability and the second leading group cause of death [1]. Over 600 types of neurological conditions have been described, with Alzheimer’s disease (AD) being the most common one (60–70% of all dementia cases). AD is characterized by a progressive and irreversible deterioration of cognitive functionality that inflicts profound harm regarding patient quality of life, posing a great challenge for carers, families, and overall society and entailing high costs to health-care systems worldwide [2].

AD is of a multifactorial nature and its pathophysiological mechanisms are still not fully understood; however, some clinical hypotheses have been postulated for setting the main neuropathological hallmarks of this condition. The cholinergic hypothesis argues that a deficit in the cholinergic neurotransmission is involved in the cognitive impairment that characterizes AD [3]. A prediction of this hypothesis is that drugs that potentiate central cholinergic function (e.g., acetylcholine (ACh) precursors, inhibitors of ACh hydrolysis, specific M_1_ muscarinic or nicotinic agonists, and M_2_ muscarinic antagonists) should improve cognition and perhaps even some of the behavioral disturbances experienced in AD [4]. The amyloid hypothesis emphasizes the presence of extracellular deposits of amyloid β-protein (Aβ) plaques in the brain as the main neuropathological hallmark of AD. Aβ peptides are generated by the sequential secretase-mediated cleavage (β- and γ-secretases) of amyloid precursor protein (APP), and the accumulation of Aβ aggregates, especially those of low molecular weight, leads to neurotoxicity [5]. Hence, Aβ formation can be hindered by targeting these secretases, which can help to delay or stop the progression of AD. The tau hypothesis acknowledges that intracellular deposits of hyperphosphorylated microtubule-associated tau protein are toxic to neurons and highly correlated with the cognitive deficits observed not only in AD but also in other neurodegenerative disorders [6]. The direct inhibition of tau aggregation has been suggested as one approach for potentially reversing neurofibrillary lesion formation [7]. The oxidative stress hypothesis has attributed a key role to the oxidative damage of biomolecules, such as lipids and proteins, that may trigger cell organelle dysfunction, ultimately leading to the demise of key-neuronal cells [8]. Owing the generally low antioxidant machinery and the high content of polyunsaturated fatty acids (PUFA) of neuronal membranes, the central nervous system (CNS) is particularly susceptible to reactive oxygen species (ROS)-mediated injury [9]. Although epidemiological data support the relationship between oxidative state and global health [10,11], the recommendation of antioxidant supplements to prevent chronic diseases still lacks evidence, and the selective nature of the blood-brain barrier limits the distribution of antioxidant molecules to the brain.

Over the past years, studies have established a strong link between neurotoxicity and an excitatory mechanism elicited by high concentrations of glutamate in the synaptic cleft, the main excitatory neurotransmitter in the mammalian CNS [12,13]. This phenomenon of excitotoxicity has been implicated in the pathophysiology of several CNS diseases, leading to neuronal dysfunction; degeneration; and, ultimately, cell death [14]. Hence, biologically active substances capable of protecting the brain cells against glutamate excitotoxicity may be a good therapeutic alternative.

During neurodegeneration, the activation of brain-resident microglia, which coordinates the immune response in the CNS, is also highly increased. If, on the one hand, neuroinflammation is acknowledged as the first line of defense against harmful stimuli, on the other hand a chronic aberrant inflammatory response contributes to neurotoxicity, oxidative stress, and synaptic and neuronal damage [15].

As the array of cellular processes leading to AD and other neurodegenerative diseases is being unveiled, it has become clear that a multitarget approach relying on the simultaneous modulation of multiple biological targets for managing physiological changes associated with neurodegeneration may represent a more realistic solution in the clinical setting to the classic “one drug, one target” paradigm [16]. So far, the pharmacotherapeutic arsenal used to fight neurological disorders cannot stop the damage to the brain from progressing, only delaying its symptomatic manifestation [17]. Besides this, they are not devoid of harmful side effects, which, together with the increasing consumer awareness and demand for bio-based products, has guided efforts towards bioprospection—i.e., the exploitation of nature diversity to find new valuable products.

Despite the long history of drug discovery from natural sources, the global marine pharmaceutical pipeline is still in its infancy. Nevertheless, several experts have considered the potential of marine-based compounds in all disease areas to be immense.

This review thoroughly addresses the main advances in a key compound class exclusively biosynthesized by brown seaweeds—i.e., phlorotannin—and its potential therapeutic targets within the complex biological scenario of neurodegeneration. Pursuing our interest on disclosing the range of biological potential of phlorotannins [18,19,20,21,22,23,24], this review aims to support and rationalize the main mechanisms underlying the neuroactive potential of these marine polyphenols.

## 2. The Marine Biosphere as a Thriving Resource of Bioactives: The Case of Phlorotannins

Oceans provide shelter for about half of the global biodiversity, creating new and exciting challenges for the scientific community. Over the last few decades, interest in the marine ecosystem has been growing, with more than 30,000 compounds having been isolated from marine organisms [25]. This remarkable diversity, together with the ability to adapt and survive in hostile environmental conditions, makes marine organisms an almost unlimited field of research with great biotechnological potential.

**Figure 1:**
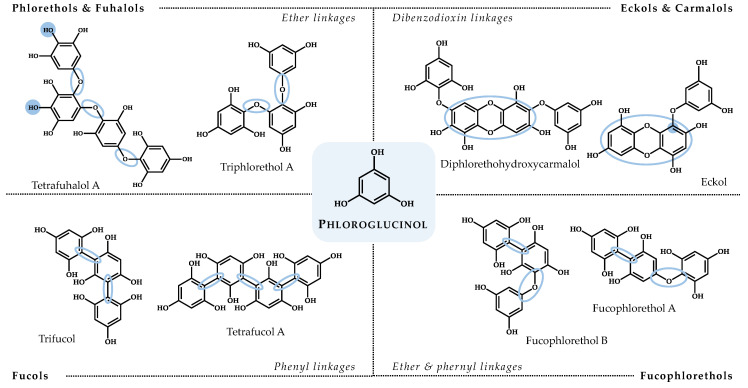
Structures of representatives of each phlorotannin class, highlighting their distinctive chemical features.

Within the marine biosphere, macroalgae (commonly addressed as seaweeds) have a vital role in supporting marine biodiversity and are widely acknowledged as prolific bio-factories of compounds [26]. Metabolites from green (Chlorophyta), brown (Ochrophyta), and red (Rhodophyta) marine algae have been addressed with several bioactivities, providing important chemical scaffolds for drug discovery and holding promise for developing novel therapeutics [27,28]. Very recently, a marine algae-derived oral oligosaccharide—sodium oligomannate (GV-971)—received its first approval for the treatment of mild to moderate AD by improving cognitive function [29]. Still, regarding neurodegenerative diseases, and despite some works having already brought to light the neuroprotective effects of compounds and extracts from macroalgae (as reviewed in [30,31,32,33,34,35]), the number of species that have been studied for neuroprotective activities is very limited, opening doors for the exploitation of several others around the globe. The latest reviews on algal-derived compounds with neuroprotective potential have highlighted a dominance of those isolated from Ochrophyta in terms of functionality [32,35]. Among the brown seaweed metabolites, special attention has been paid to phlorotannins. Phlorotannins are chemically categorized as phloroglucinol (1,3,5-trihydroxybenzene)-based polyphenols and are structurally distinguished according to the type of linkage between the phloroglucinol monomers and the number and distribution of hydroxyl (OH) groups in their molecular backbone. Summarily, phlorotannins can be classified as follows: (i) phlorethols (aryl-ether bonds), (ii) fuhalols (ortho- and para-arranged ether bonds with an additional OH group), (iii) fucols (aryl-aryl bonds), (iv) fucophlorethols (ether and phenyl linkages), (v) eckols (dibenzodioxin elements substituted by a phenoxyl group at C-4), and (vi) carmalols (derivatives of phlorethols with a dibenzodioxin moiety) [36] (Figure 1).

Besides playing key roles in different stages of the development of brown seaweeds and acting as algal chemical defenses, a range of bioactive properties have also been reportedly attributed to phlorotannins (as reviewed in [37]).

Under experimental conditions phlorotannins and phlorotannins-rich extracts/fractions have displayed positive health-related effects, including antioxidant [38,39], antimicrobial [20,40], anti-hyperglycemic [23,41], antiproliferative [42,43], anti-inflammatory [19,21,44], anti-allergic [22,45], and neuroactive effects [46,47].

Though research on the neuroprotective properties of phlorotannins is still scarce, these brown seaweed polyphenols have been pointed out as promising candidates for the development of new generation disease-modifying agents to address the challenge of neurodegeneration.

### 2.1. Neuroactive Potential of Phlorotannins: Evidence from In Vitro and In Vivo Studies

**Figure 2:**
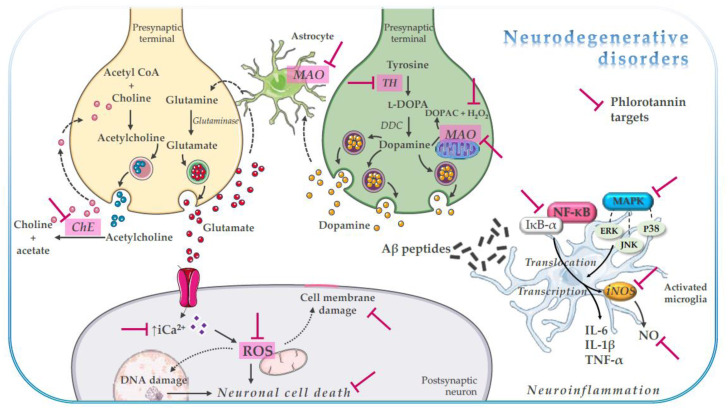
Schematic representation of phlorotannin multimodal neuroactivity. Aβ, amyloid-β; Ca^2+^, calcium; ChE, cholinesterase; DOPAC, 3,4-dihydroxyphenylacetic acid; ERK, extracellular signal-regulated kinase; IL, interleukin; IkB-α, inhibitory kB-α; JNK, c-Jun N-terminal kinase; MAPK, mitogen-activated protein kinase; MAO, monoaminoxidase; NF-kB, nuclear factor-kB; NO, nitric oxide; ROS, reactive oxygen species; TNF-α, tumor necrosis factor-α; TH, tyrosine hydroxylase.

Phlorotannins have been found to exert their neuroprotective effects through multimodal action (Figure 2, Table 1 and Table 2), as evidenced in in vitro studies by their capacity to inhibit CNS-related enzymes [24,48,49,50,51,52,53,54,55,56,57,58,59,60,61], modulate neuronal receptors [52,62], and regulate signaling pathways linked to oxidative stress-mediated neuronal cell death [24,63,64,65,66,67,68,69,70,71,72] and neuroinflammation [73,74,75,76,77].

#### 2.1.1. Modulation of CNS-Related Enzymatic Targets

Enzymes have emerged as critical regulators of neurodegenerative diseases and it has been clinically demonstrated that modulating the activity of key enzymes, though neither slowing down nor blocking basic pathological mechanisms, can afford symptomatic relief.

Among their most promising biological features, phlorotannins can associate with proteins to form enzyme-inhibitor complexes [78], granting them the potential to interact with enzymes involved in many pathophysiological processes.

##### Acetyl- and Butyrylcholinesterases

Although generally recognized to be a pathological hallmark of AD, cholinergic denervation is also shared by other neurological disorders, and it leads to a decline in acetylcholine (ACh) levels in the brain [79]. Acetylcholinesterase (AChE) and butyrylcholinesterase (BChE), though differing in substrate specificity, kinetics, and activity in different brain regions, are two key enzymes involved in the regulation of Ach levels. Hence, the first-line therapy has relied on the use of cholinesterase (ChE) inhibitors to retard the inactivation of ACh after synaptic release and improve cognitive function [80].

In spite of several ChE inhibitors having been isolated from terrestrial natural origin, research on effective anti-ChE agents from marine algae is comparatively scarce [81]. The ChE inhibitory capacity of phlorotannins has been mainly devoted to compounds of eckol class [49,50,54,58,59]. In the study conducted by Yoon et al. [49], eckstolonol, eckol, dieckol, 2-phloroeckol, and 7-phloroeckol, isolated from *Ecklonia stolonifera* Okamura, showed a selective dose-dependent inhibitory activity towards AChE over BChE [49]. Conversely, Choi et al. [59] found that phlorofucofuroeckol-A isolated from *Ecklonia cava* Kjellman was particularly potent at inhibiting BChE (IC_50_ = 0.95 μM), with an activity over 100-fold higher than AChE inhibition [59]. In a recent work conducted by our group [24], strong correlations were found between the amount of phlorotannins and both the AChE and BChE inhibitory capacity of targeted extracts obtained from different *Fucus* species, harvested along the Portuguese coastline [24]. It was also demonstrated that the extracts with a higher phlorotannin content were selectively more active towards AChE than against BChE [24]. Differences in terms of enzyme selectivity may be the result of the specific binding properties between the enzyme and substrate [49]; however, the degree of polymerization and other structural features of the phlorotannin backbone play important roles in the inhibitory potential of phlorotannins against ChEs [49,50,54].

8,8′-Bieckol was one of the most potent AChE inhibitors (IC_50_ = 4.59 μM) [50]. The AChE inhibition kinetics indicated that 8,8′-bieckol acted as a competitive inhibitor, interacting directly with the catalytic site of the enzyme [50]. Interestingly, 6,6′-bieckol, a positional isomer of 8,8′-bieckol, was found to be a non-competitive inhibitor for the hydrolysis of ACh catalyzed by AChE, altering the structure of the enzyme, which is no longer able to bind with a substrate correctly [54].

##### Monoaminoxidases

Alongside ChE, evidence have also pointed to the relevance of monoaminoxidases (MAO) in key pathophysiological mechanisms in AD and other neurodegenerative diseases, leading to cognitive dysfunction, the destruction of cholinergic neurons, and the formation of amyloid plaques [16]. Though sharing structural and functional similarities, the two human isoforms of MAO (MAO-A and MAO-B) differ from each other by substrate specificity [82]. MAO-A metabolizes serotonin, a neurotransmitter implicated in depression, while MAO-B catalyzes the oxidation of aminyl substrates, such as dopamine, whose levels are generally diminished in patients with Parkinson’s disease (PD), as result of the progressive degeneration of neurons of the substantia nigra. While MAO-B inhibitors are currently used in the clinical setting for the early symptomatic treatment of PD, little attention has been paid to the potential disease-modifying effects of MAO-A inhibitors. However, MAO-A suppression, as has been suggested for MAO-B inhibition, might also provide neuroprotective effects, mainly related to the attenuation of oxidative stress [83].

Only recently has the MAO inhibitory potential of isolated phlorotannins and phlorotannin-rich extracts been evaluated [24,52,60]. In the study of Jung et al. [60], eckol and dieckol isolated from *Eisenia bicyclis* (Kjellman) Setchell inhibited both MAO isoenzymes, showing relative selectivity towards MAO-A over MAO-B (Selectivity index (*SI*)_eckol_ = 0.09 vs. *SI*_dieckol_ = 0.26) [60]. While eckol (a trimer of phloroglucinol) was more potent at inhibiting MAO-A (IC_50(MAO-A)_ = 7.20 μM vs. IC_50(MAO-B)_ = 11.43 μM) than dieckol (an hexamer of phloroglucinol), the latter exhibited a higher inhibitory activity towards MAO-B (IC_50(MAO-B)_ = 43.42 μM vs. IC_50(MAO-A)_ = 83.44 μM) [60]. Eckol displayed a mixed-type inhibition of MAO-A, and it acted as a non-competitive inhibitor on MAO-B; dieckol, on the other hand, showed a non-competitive inhibitory mechanism towards both MAO isoforms [60]. Although no specific residues were found to be responsible for inhibiting MAO, eckol and dieckol displayed very different binding behaviors, mainly linked to the number of OH groups in their molecular backbone that increased the tendency of hydrogen bond interaction [60]. In the latter work by Seong et al. [52], phlorofucofuroeckol-A showed a significant inhibitory effect on both MAO isoenzymes (IC_50(MAO-A)_ = 9.22 μM vs. IC_50(MAO-B)_ = 4.89 μM), with higher selectivity towards MAO-B (*SI* = 1.89). This pentamer of phloroglucinol was found to bind to the surface of both MAO isoforms, at non-catalytic sites, which is consistent with a non-competitive mechanism of action [52].

Besides inhibiting MAO, eckol, dieckol, and phlorofucofuroeckol-A were found to be agonists of dopamine D_3_/D_4_ receptors [52,62]. However, only dieckol and phlorofucofuroeckol-A acted as antagonists of D_1_ receptor and had effects in other receptors that also play important roles in the regulation of emotional behavior (e.g., muscarinic acetylcholine (M_5_), neurokinin-1 (NK_1_), serotonin (5-HT_1A_), and vasopressin (V_1A_) receptors). Seong et al. [52] established a structure-activity relationship (SAR) between the tested phlorotannins and the target proteins, including MAO and G-couple protein receptors (GCPRs): (i) more than three phloroglucinol units (PGU) are required to inhibit MAO and D_3_/D_4_ receptors, and (ii) more than five PGU are essential for the inhibition of D_1_, NK_1_, and 5-HT_1A_ receptors [52]. In fact, in the work by Barbosa et al. [24], the compounds behind the multifunctionality of phlorotannin-targeted extracts from *Fucus* spp. on in vitro targets underpinning neurodegeneration, including the modulation of the activity of MAO-A and MAO-B, were those with more than three PGU, in agreement with the SAR studies by Seong et al. [52].

##### β-Secretase

Insoluble Aβ, which aggregates into oligomers and fibrils leading to the plaque deposition and neurodegeneration, result from the sequential cleavage of APP catalyzed by β- and γ-secretase [5]. Blocking the activity of the enzymes involved in the production of Aβ-protein, especially the β-site APP cleaving enzyme (BACE-1), has been considered as one of the most attractive anti-amyloid strategies for tackling AD. The search for potent BACE-1 inhibitors has been a hard task, and many compounds have failed to prosecute clinical trials [84]. The first research works addressing the BACE-1 inhibitory capacity of phlorotannins have emerged during the last decade [50,55,57,61].

Jung et al. [55] demonstrated anti-BACE-1 inhibitory potential of four eckol-type phlorotannins (dioxinodehydroeckol, eckol, phlorofucofuroeckol-A, dieckol, and 7-phloroeckol) and of a phlorethol derivative (triphlorethol-A) isolated from the edible perennial brown seaweed *E. bicyclis*. Most of the studied phlorotannins were found to inhibit BACE-1 in a non-competitive manner, with phlorofucofuroeckol-A and dieckol having been not only the most potent compounds (IC_50_ values of 2.13 µM and 2.21 µM, respectively), but also the more effective inhibitors (inhibition constants (*Ki*) of 1.3 and 1.5, respectively) [55]. Likewise, fucofuroeckol-B, isolated from the seaweed species *E. bicyclis*, was found to effectively inhibit BACE-1 activity (IC_50_ = 16.1 µM) by either binding with the enzyme or with the enzyme-substrate complex [61].

The phloroglucinol hexamer 8,8′-bieckol, isolated from *E. cava*, displayed a strong BACE-1 inhibition (IC_50_ = 1.62 µM), also acting as non-competitive inhibitor [50]. In fact, the docking results showed that H-bonds between 8,8′-bieckol and allosteric residues of BACE-1 play a key role in enzyme inhibition [50], and it has also been hypothesized that the steric hinderance of the OH and aryl groups near the biaryl linkage of 8,8′-bieckol is responsible for promoting its generally higher inhibitory potency [50,85].

##### Tyrosinase

Tyrosinase is a multifunctional copper-containing enzyme that controls the synthesis of melanin in a two-step process, acting as (i) a monophenolase, hydroxylating monophenols such as l-tyrosine, and as (ii) a diphenolase, oxidizing *o*-diphenols to the corresponding *o*-quinones, which undergo several reactions leading to melanin [86]. The works addressing the anti-tyrosinase capacity of phlorotannins, focused mainly on their potential application in the cosmetic industry for managing skin conditions related to hyperpigmentation. However, tyrosinase inhibitors have also been explored for food and medicinal applications, namely in neurodegenerative diseases [87]. Aside from participating in the synthesis of peripheral melanin, it was recently found that the overexpression of tyrosinase in the substantia nigra results in the accumulation of neuromelanin up to levels that may interfere with normal cell function and trigger Parkinson-like neuronal dysfunction/degeneration [88]. Besides this, there is a selective degeneration of neuromelanin-containing neurons in PD [88], which makes the inhibition of tyrosinase activity a very promising approach to prevent, halt, or delay neurodegenerative processes.

The monomer phloroglucinol itself, together with four phloroglucinol derivatives (eckstolonol, eckol, phlorofucofuroeckol-A, and dieckol), isolated from *E. stolonifera*, inhibited tyrosinase activity. Among the isolated compounds, dieckol showed a three times higher inhibitory power than the reference standard, kojic acid, and acted as a non-competitive inhibitor of tyrosinase [48]. Besides directly inhibiting the activity of mushroom tyrosinase, dieckol also reduced the melanin content in α-melanocyte stimulating hormone (α-MSH)-elicited B16F10 melanoma cells, by the inhibition of murine tyrosinase, more effectively than the commercial agent arbutin [56].

In a more recent study, Kim et al. [53] disclosed a time-dependent inhibition of tyrosinase by 2-phloroeckol and 2-*O*-(2,4,6-trihydroxyphenyl)-6,6′-bieckol, isolated from *E. cava*, showing the characteristics of slow-binding inhibitors [53]. Manandhar et al. [51] demonstrated, for the first time, the potent anti-tyrosinase potential of an octamer of phloroglucinol (974-A), as well as its capacity to reduce the cellular melanin content and to downregulate the expression of melanogenic enzymes (tyrosinase, tyrosinase-related protein (TRP)-1, and TRP-2) in an α-MSH-induced B16F10 melanoma cells [51]. The authors also unveiled, through docking molecular simulations, the relevance of the OH moiety in exerting the anti-tyrosinase activity, as most of the OH groups of the isolated phlorotannins formed H bonds with tyrosinase residues, both at the catalytic and allosteric sites of the enzyme [51].

Phlorotannins present in targeted extracts from *Fucus* spp. were also found to have a preferential binding to wide regions of the enzyme other than to the active site, behaving as non-competitive inhibitors of tyrosinase [24].

#### 2.1.2. Attenuation of Cell Neurotoxicity

Neurotoxicity (i.e., damage to the brain or the central and peripheral nervous systems triggered by biological or physicochemical agents) has been implicated in brain ischemia/stroke, traumatic brain injury, and neurodegenerative diseases [89]. In particular, neurotoxicity may be induced by an imbalance in antioxidant defense systems, accompanied by an overload of oxidizing species that lead to the phenomenon commonly addressed as oxidative stress.

Different agents, including hydrogen peroxide (H_2_O_2_), rotenone, glutamate, and Aβ oligomers, have been employed as neurotoxic challenge paradigm to evaluate and characterize the effects of phlorotannins in different aspects of neuroprotection [24,63,64,65,66,67,68,69,70,71,72].

Phloroglucinol and the phlorotannins eckol, triphorethol-A, eckstolonol, and dieckol, isolated from *E. cava*, were able to protect murine hippocampal HT22 cells against H_2_O_2_-induced neurotoxicity by (i) the suppression of intracellular ROS, (ii) the inhibition of cell membrane peroxidation, and (iii) the reduction in apoptotic events, such as nuclear fragmentation and intracellular Ca^2+^ levels [64].

Among the phlorotannin compounds studied so far, phlorofucofuroeckol-A demonstrated a strong potential to interact with Aβ peptides, preventing their self-assembly and therefore inhibiting Aβ aggregation [69]. In fact, in a previous report by Ahn et al. [70], the anti-amyloidogenic activity of this phloroglucinol pentamer was disclosed and it was hypothesized that the neuroprotective effects may be mediated through reduced intracellular ROS and Ca^2+^ generation [70]. Phlorofucofuroeckol-A also protected PC12 cells from glutamate-induced neurocytotoxic damage, through the attenuation of caspase-dependent apoptosis cell death, the regulation of cytosolic and mitochondrial ROS generation, and the improvement of mitochondrial disfunction mediated by rescuing membrane potential (ΔΨm) and mitochondrial mass [71]. Likewise, dieckol protected both primary cortical neurons and HT22 cells against glutamate toxicity-induced cell death and morphological deterioration by a reduction in ROS levels, the attenuation of mitochondrial disfunction, and the activation of the nuclear factor-like 2/heme oxygenase-1 (Nrf-2/HO-1) pathway as a cellular antioxidant defense system [63]. Dieckol was also found to reduce the intracellular ROS and cytochrome *c* release on rotenone-induced neurotoxocity and α-synuclein aggregation in a neuroblastoma cell line (SH-SY5Y) [65].

Eckmaxol, a hexamer of phloroglucinol isolated from *Ecklonia maxima* (Osbeck) Papenfuss, exhibited anti-amyloidogenic activity in SH-SY5Y cells, preventing but not rescuing Aβ oligomer-induced neuronal apoptosis and an increase in intracellular ROS [72]. The neuroprotective potential of eckmaxol was attributed to the regulation of glycogen synthase kinase (GSK) 3β, which has been proposed to be a critical molecular link between the extracellular Aβ plaques and the intracellular neurofibrillary tangles formed from hyperphosphorylated tau protein [72]. In fact, the phlorotannins eckol, dieckol, 6,6′-bieckol, 8,8′-bieckol, and phlorofucofuroeckol-A have previously been found to interact with GSK3β [59].

Besides inhibiting the BACE-1 activity, fucofuroeckol-B exhibited neuroprotective effects against β-amyloid toxicity by reducing the BACE-1-catalyzed cleavage of APP and Aβ generation in a transgenic human neuroblastoma cell line (SH-SY5Y-APP695swe) [61].

In addition to the studies addressing the neuroprotective effects of single isolated phlorotannin components, research has been also highlighting the superior effectiveness of phlorotannin extracts/fractions to counteract cell neurotoxicity [24,66,67,68]. Barbosa et al. [24] have suggested that the neuroactive potential of phlorotannin-targeted extracts from *Fucus* spp. results from synergistic interactions between the phlorotannins present thereof and emphasize the use of targeted extracts over that of isolated compounds [24]. Although none of the *Fucus* spp. phlorotannin extracts significantly restored the viability of glutamate-damaged SH-SY5Y cells, no cytotoxicity exacerbation was observed in cells treated with the extracts and co-exposed to glutamate. Furthermore, the phlorotannin extract from *Fucus serratus* Linnaeus, which was amongst the richest in terms of total phlorotannin content and that displayed high total antioxidant capacity, was indeed the most promising extract at attenuating oxidative glutamate toxicity in SH-SY5Y cells [24].

The works by Alghazwi et al. [66] and Shrestha et al. [67] have both demonstrated the neuroprotective and anti-Aβ aggregatory properties of phlorotannin-rich extracts from *Ecklonia radiata* (C.Agardh) J.Agardh, with the latter ascribing the effects to the dominant presence of eckol-type phlorotannins [67].

A phlorotannin-rich extract from *E. cava* and its main component, dieckol, were found to protect neuronal PC12 and SH-SY5Y cells from intracellular oxidative stress partly due to their antioxidant properties [68]. However, treatment with *E. cava* extract, but not with dieckol, led to an increase in the neuronal cell viability, reinforcing the relevance of other phlorotannin components in the extract to the neuroprotective effects [68].

#### 2.1.3. Anti-Neuroinflammatory Properties

Excessive activation of microglia, a specialized form of resident macrophages in the brain, and subsequent neuroinflammation result in synaptic loss and disfunction. Hence, mechanisms to regulate microglial activation may reduce neuronal injury or death in neurodegenerative diseases. During the last decade, the anti-neuroinflammatory potential of phlorotannins has been a hot topic of research [73,74,75,76,77]. Overall, studies have looked at the capacity of phlorotannins to act upon different critical steps of inflammatory response, resorting to the in vitro model of BV2 microglia cells, following activation with the well-known bacterial endotoxin lipopolysaccharide (LPS), a potent elicitor of pro-inflammatory cytokines and inflammation mediators’ production [73,74,75,76].

Dieckol effectively decreased the LPS-induced cytokine production, acting at the transcriptional level, by the suppression of inducible nitric oxide synthase (iNOS) and cyclooxygenase (COX)-2 expression [73]. The anti-neuroinflammatory properties of dieckol were found to be mediated by blockade of nuclear factor (NF)-κB and p38 mitogen-activated protein kinases (MAPK) activation, as well as by displaying antioxidant effects in BV2 microglia [73]. Thereafter, it was demonstrated that dieckol suppresses microglia-mediated neurotoxicity implicated in the pathogenesis of neuroinflammation and neurodegeneration via suppression of microglial activation, which is mediated by the downregulation of extracellular signal-regulated kinase (ERK), phosphoinositide-3-kinase-protein kinase B (PI3K-PKB/Akt) and nicotinamide adenine dinucleotide phosphate (NADPH) oxidase pathways [75]. In a more recent study, conducted by Lee et al. [77], dieckol was amongst the most active phlorotannins at displaying anti-neuroinflammatory properties related to the downregulation of pro-inflammatory enzymes, by suppressing NF-κB and MAPK activation [77].

Similarly to what has been disclosed for dieckol, the phlorotannins phlorofucofuroeckol-B and 6,6′-bieckol were both found to exert their anti-neuroinflammatory effects mainly by the downregulation of the NF-κB and MAPK pathways, accompanied by a sharp decrease in cytokine production and in the expression of pro-inflammatory proteins [74,76].

#### 2.1.4. From In Vitro Potential to In Vivo Assessment of Phlorotannin Neuroactivity

To date, only a few in vivo studies have addressed the neurological activity of phlorotannins and phlorotannin preparations, but their somnogenic [47,90,91,92] and memory-enhancing [46,93,94,95] effects have already been demonstrated.

Myung et al. [46] found that the repeated administration of either dieckol or phlorofucofuroeckol reduced the ethanol-induced latency inhibition in mice and regulated the levels of some central neurotransmitters, especially increasing those of ACh in the striatum, hippocampus, and cortex by the inhibition of AChE activity. Phlorofucofuroeckol was also found to act as neuroprotective agent in ischemic stroke by significantly reducing coronal infarct volume (more than 70%) and severe cellular responses in vivo (e.g., neuronal shrinkage, and apoptosis) in a middle cerebral artery occlusion (MCAO) model [71].

Yang et al. [94] demonstrated that the stereotaxic injection of phloroglucinol, the phlorotannins’ building block, attenuated cognitive function impairments in the 5XFAD mouse model of AD by regulating synaptic plasticity, with the reduction in dendritic spine density and the levels of synaptic proteins ((synaptophysin and post synaptic density protein 95 (PSD-95)) [94]. In a later work by Yang et al. [95], the oral administration of phloroglucinol also attenuated the cognitive deficits in 5XFAD mice and, besides restoring dendritic spine density, a significant reduction in the number of Aβ plaques and in the protein level of BACE-1 was observed. In addition, phloroglucinol prevented lipid peroxidation, slowed down the reactivation of glial cells, and reduced the release of pro-inflammatory cytokines in 5XFAD mice [95].

The effects of phloroglucinol were also evaluated in an in vivo experimental model of PD, using 6-hydroxydopamine (6-OHDA) as a neuronal damage inductor [96]. The authors found that phloroglucinol improved 6-OHDA-induced-motor functional deficits, also acting as protective agent against the loss of dopaminergic neurons and, consequently, the reduction in synapses between dopaminergic neurons in the midbrain [96]. Mechanistically, phloroglucinol was capable of (i) restoring the reduction in Nrf2 in the nuclear fraction induced by 6-OHDA treatment, and (ii) reversing the 6-OHDA-mediated loss of the activity and expression of the antioxidant enzymes catalase and glutathione peroxidase [96].

**Table 1:** Summary of the neuroactive properties of phloroglucinol (monomeric unit) and isolated phlorotannins ^1^.

Compound	Seaweed Species	Experimental Model	Proposed Mechanism of Action	Reference(s)
974-A	*Ecklonia stolonifera* Okamura	Cell-free enzymatic system α-MSH-elicited B16F10 cells	Tyrosinase inhibition ↓ Tyrosinase, TRP-1, TRP-2 expression	[51]
6,6′-Bieckol	*Ecklonia cava* Kjellman *Ishige okamurae* Yendo	Cell-free enzymatic system LPS-stimulated BV2 and primary microglial cells	AChE, BChE, and BACE-1 inhibition ↓ NO, PGE_2_, TNF-α, IL-1β, and IL-6 levels ↓ iNOS and COX-2 expression ↓ NF-kB activation ↓ Akt, JNK, and p38 MAPK phosphorylation	[54,59,76]
6,8′-Bieckol	*E. cava*	Cell-free enzymatic system	Tyrosinase inhibition	[53]
8,8′-Bieckol	*E. cava*	Cell-free enzymatic system Aβ-induced PC12 cells	AChE, BChE, BACE-1, and tyrosinase inhibition ↓ Cell death ↓ NO and PGE_2_ levels ↓ iNOS, COX-2, TNF-α, and IL-1β expression ↓ NF-kB activation ↓ JNK and p38 MAPK phosphorylation	[50,53,59,77]
2-*O*-(2,4,6-trihydroxyphenyl)-6,6′-bieckol	*E. cava*	Cell-free enzymatic system	Tyrosinase inhibition	[53]
2-Phloroeckol	*E. stolonifera* *E. cava*	Cell-free enzymatic system	AChE and tyrosinase inhibition	[49,53]
7-Phloroeckol	*E. stolonifera* *Eisenia bicyclis* (Kjellman) Setchell	Cell-free enzymatic system Aβ-induced PC12 cells	AChE and BACE-1 inhibition ↓ Cell death ↓ ROS and Ca^2+^ levels	[49,55,70]
Dieckol	*E. stolonifera* *E. cava* *E. bicyclis*	Cell-free enzymatic system Cell-based functional assays with stable cell lines expressing recombinant GPCRs α-MSH-elicited B16F10 cells H_2_O_2_-induced HT22 cells H_2_O_2_- and AAPH-induced SH-SY5Y and PC12 cells Aβ-induced PC12 cells Glutamate-induced HT22 cells and primary cortical neurons Rotenone-induced SH-SY5Y cells LPS-stimulated BV2 cells Ethanol-treated mice	AChE, BChE, BACE-1, MAO-A, MAO-B, and tyrosinase inhibition D_1_R, NK_1_, and 5-HT_1A_ antagonism D_3_R, D_4_R, and V_1A_ agonism ↓ Lipid peroxidation ↓ Nuclear fragmentation ↓ Cell death ↓ ROS, Ca^2+^, NO, PGE_2_, TNF-α and IL-1β levels ↓ iNOS, COX-2, TNF-α, and IL-1β expression ↑ gp91*^phox^* expression ↓ ERK and Akt phosphorylation ↓ NF-kB activation ↓ Mitochondrial disfunction ↑ Nrf2/HO-1 activation ↓ Cytochrome *c* release ↓ α-synuclein aggregation ↓ Learning acquisition inhibition Regulation of neurotransmitter levels	[46,48,49,50,52,55,56,59,60,63,64,65,68,70,73,75,77]
Dioxinodehydroeckol	*E. bicyclis*	Cell-free enzymatic system	BACE-1 inhibition	[55]
Diphlorethohydroxycarmalol	*I. okamurae*	Cell-free enzymatic system	BChE inhibition	[54]
Eckol	*E. stolonifera* *E. cava* *E. bicyclis*	Cell-free enzymatic system Cell-based functional assays with stable cell lines expressing recombinant GPCRs α-MSH-elicited B16F10 cells H_2_O_2_-induced HT22 cells Aβ-induced PC12 cells	AChE, BChE, BACE-1, MAO-A, MAO-B, and tyrosinase inhibition D_3_R and D_4_R agonism ↓ Tyrosinase, TRP-1, TRP-2, iNOS, COX-2, TNF-α, and IL-1β expression ↓ ROS, Ca^2+^, NO, and PGE_2_ levels ↓ Lipid peroxidation ↓ Nuclear fragmentation ↓ Cell death ↓ NF-kB activation ↓ p38 MAPK phosphorylation	[48,49,50,51,52,53,55,59,60,64,70,77]
Eckmaxol	*Ecklonia maxima* (Osbeck) Papenfuss	Aβ-induced SH-SY5Y cells	↓ Cell death ↓ ROS levels GSK3β and MEK inhibition	[72]
Eckstolonol	*E. stolonifera*	Cell-free enzymatic system H_2_O_2_-induced HT22 cells	AChE and tyrosinase inhibition ↓ ROS and Ca^2+^ levels ↓ Lipid peroxidation ↓ Nuclear fragmentation	[48,49,64]
Fucofuroeckol-B	*E. bicyclis*	Cell-free enzymatic system	BACE-1 inhibition	[61]
Phlorofucofuroeckol	*E. cava*	Ethanol-treated mice Glutamate-stimulated PC12 cells MCAO-induced in rats	↓ Learning acquisition inhibition Regulation of neurotransmitter levels ↓ Caspase-dependent apoptosis ↓ ROS levels ↓ Mitochondrial damage ↓ Coronal infarct volume ↓ Severe cellular responses	[46,71]
Phlorofucofuroeckol-A	*E. cava* *E. stolonifera* *E. bicyclis*	Cell-free enzymatic system Cell-free non-enzymatic system Cell-based functional assays with stable cell lines expressing recombinant GPCRs α-MSH-elicited B16F10 cells Aβ-induced PC12 cells	AChE, BChE, BACE-1, and tyrosinase inhibition Aβ_25–35_ self-aggregation inhibition D_1_R, NK_1_, and 5-HT_1A_ antagonism D_3_R and D_4_R agonism ↓ Tyrosinase, TRP-1, and TRP-2 expression ↓ Cell death ↓ ROS and Ca^2+^ levels	[48,51,52,53,55,59,69,70]
Phlorofucofuroeckol-B	*E. stolonifera*	LPS-stimulated BV2 cells	↓ NO, PGE_2_, TNF-α, IL-1β, and IL-6 levels ↓ iNOS and COX-2 expression ↓ NF-kB pathway ↓ Akt, ERK and JNK phosphorylation	[74]
Phloroglucinol	*E. bicyclis* *E. stolonifera* *E. cava*	Cell-free enzymatic system H_2_O_2_-induced HT22 cells Aβ-induced PC12 cells Glutamate-stimulated SH-SY5Y cells Aβ-induced HT22 cells Aβ-induced primary hippocampal neuron cultures 6-OHDA-induced SH-SY5Y cells 5XFAD mice 6-OHDA-lesioned rats	BACE-1 and tyrosinase inhibition ↓ Cell death ↓ Nuclear fragmentation ↓ ROS and Ca^2+^ levels ↓ Lipid peroxidation, protein carbonylation, and DNA base modification ↓ BACE-1, GFAP, Iba-1, TNF-α, and IL-6 expression ↑ Catalase and glutathione peroxidase activity and expression ↑ Nrf2 activation ↓ Cognitive and motor function impairments ↓ Dopaminergic neurons and synapse loss ↓ Aβ plaques ↑ Dendritic spine density and mature spines ↑ Synaptophysin and PSD-95 expression	[24,48,55,64,70,94,95,96]
Triphlorethol-A	*E. bicyclis* *E. cava*	Cell-free enzymatic system H_2_O_2_-induced HT22 cells	BACE-1 and tyrosinase inhibition ↓ ROS and Ca^2+^ levels ↓ Lipid peroxidation ↓ Nuclear fragmentation	[53,55,64]

^1^ 5-HT_1A_, serotonin 1A receptor; 6-OHDA, 6-hydroxydopamine; α-MSH, α-melanocyte-stimulating hormone; Aβ, amyloid β-protein; AAPH, 2,2′-azobis(2-amidinopropane) dihydrochloride; AChE, acetylcholinesterase; Akt, protein kinase B; BACE-1, β-site amyloid precursor protein cleaving enzyme 1; BChE, butyrylcholinesterase; COX-2, cyclooxygenase-2; D_1_R, dopamine D_1_ receptor, D_3_R, dopamine D_3_ receptor; D_4_R, dopamine D_4_ receptor; ERK, extracellular signal-regulated kinase; GFAP, glial fibrillary acidic protein; GPCRs, G protein-coupled receptors; GSK3β, Glycogen synthase kinase 3β; Iba-1, allograft inflammatory factor 1; IL, interleukin; iNOS, inducible nitric oxide synthase; JNK, c-Jun N-terminal kinase; LPS, lipopolysaccharide; MAO-A, monoaminoxidase-A; MAO-B, monoaminoxidase-B; MAPK, mitogen-activated protein kinase; MCAO, middle cerebral artery occlusion; MEK, MAPK kinase; NF-kB, nuclear factor- κB; NK_1_, neurokinin 1 receptor; NO, nitric oxide; Nrf2/HO-1, nuclear factor erythoid-2-related factor 2/heme oxygenase-1; PGE_2_, prostaglandin E_2_; PSD-95, postsynaptic density protein 95; ROS, reactive oxygen species; TNF-α, tumor necrosis factor-α; TRP, tyrosinase-related protein; V_1A_, vasopressin V_1A_ receptor.

**Table 2:** Summary of the neuroactive properties of phlorotannin-rich extracts/fractions obtained from brown seaweeds.

Seaweed Species	Extract/Fraction	Experimental Model	Proposed Mechanism of Action	Reference(s)
*Fucus guiryi* Zardi, Nicastro, E.S. Serrão & G.A. Pearson *Fucus serratus* Linnaeus	Acetone:water (7:3, *v*/*v*) extract purified with microcrystalline cellulose	Cell-free enzymatic system Glutamate-stimulated SH-SY5Y cells	AChE, BChE, MAO-A, and tyrosinase inhibition ↓ Lipid peroxidation ↓ Glucose-, fructose-, and methylglyoxal-mediated protein glycation ↓ ROS levels	[24]
*Fucus spiralis* Linnaeus *Fucus vesiculosus* Linnaeus	Acetone:water (7:3, *v*/*v*) extract purified with microcrystalline cellulose	Cell-free enzymatic system Glutamate-stimulated SH-SY5Y cells	Tyrosinase inhibition ↓ Lipid peroxidation ↓ Fructose-mediated protein glycation ↓ ROS levels	[24]
*Ecklonia cava* Kjellman	Ethanol (50%, *v*/*v*) extract	H_2_O_2_- and AAPH-induced PC12 and SH-SY5Y cells	↓ ROS levels	[68]
*Ecklonia radiata* J.Agardh	Ethanol (90%, *v*/*v*) extract Ethyl acetate fraction of ethanol (80%, *v*/*v*) extract	Aβ-induced PC12 cells	↓ Cell death ↓ Aβ_1–42_ aggregation ↑ Neurite outgrowth	[66,67]

Aβ, amyloid β-protein; AAPH, 2,2′-azobis(2-amidinopropane) dihydrochloride; AChE, acetylcholinesterase; BChE, butyrylcholinesterase; MAO-A, monoaminoxidase-A; ROS, reactive oxygen species.

### 2.2. Addressing Phlorotannin Bioavailability and Blood-Brain Barrier (BBB)-Crossing Ability

Seaweeds have been an important part of the human diet all around the globe: in Pacific and Asian cultures, seaweeds have long been consumed in a variety of dishes; in Europe, the traditional consumption of seaweed-based foods has been limited to a few countries, such as Iceland, Wales, and France, but recent trends have shown an increasing acceptance of seaweeds in the Western diet [97,98]. Although epidemiological data concerning the effects of seaweed consumption are still scarce, studies comparing Asian and Western diets show an association between seaweed consumption and a lower incidence of chronic diseases (as reviewed in [99]). Regarding neurological disorders, a cross-sectional study conducted by Miyake et al. [100] found that seaweed consumption may be inversely associated with the prevalence of depressive symptoms during pregnancy [100].

Dietary habits are indeed the major source of polyphenols, and it has been reported that the consumption of brown seaweeds is on average around 1.3 kg per person, per year, containing nearly 5% of phlorotannins [101].

To reach their targets, dietary polyphenols must endure physicochemical alterations in the gastrointestinal tract, where they act as substrates for several enzymatic systems and are biotransformed [102]. Regarding phlorotannins, their complexity and the lack of commercially available analytical standards are the main limitations for bioavailability studies, leading to possible quantification errors as phloroglucinol equivalents and to a limited capability for method development, especially for the analysis of biological samples [101]. In general, it was found that phlorotannins with a high molecular weight (HMW) (>10 kDa) were poorly absorbed in the small intestine, but they were subjected to phase II conjugation reactions with the formation of glucuronides and sulphates [101]. More transformations occur in the large intestine, with a high colonic fermentation of HMW phlorotannins into phlorotannin oligomers, some of which were detected in the urine of healthy volunteers [101]. Obviously, the main objective of these kind of studies is to understand if the effects observed in vitro for the isolated compounds remain the same or can be extrapolated for an in vivo situation. It was found then that IL-8, an important inflammatory mediator, is a possible target for phlorotannin metabolites [101]. In a later study by Corona et al. [103], digested phlorotannins were reported to inhibit the growth of human colorectal adenocarnimoma HT-29 cells, while those resulting from colonic fermentation showed an antigenotoxic potential, counteracting the DNA damage caused by a pro-oxidant stimulus [103].

When the CNS is the ultimate goal, polyphenols such as phlorotannins have to cross over its physical defenses: the BBB that separates the circulating blood from the brain extracellular fluid. To date, information on phlorotannin availability in brain cells, via oral or systemic introduction, is still scarce and a major limitation to fully understanding their neuroactivity and mechanism of action in vivo. Although phlorotannin’s action on gamma aminobutyric acid type A (GABAA)-benzodiazepine receptors has been demonstrated [47], supporting their BBB-crossing ability, as far as we know only dieckol has been effectively shown to successfully penetrate the brain by BBB via still unknown transportation mechanisms [104]. Eckol, though by in silico pharmacokinetic parameter prediction, has also been suggested to have favorable drug-like properties [62]. Nevertheless, the mediated transport across the BBB through novel drug delivery systems to enhance phlorotannin delivery while ensuring the inherent bioactivities holds great promise for a non-invasive therapeutic tool and represents a valuable research opportunity.

## 3. Conclusions

Compared to the number of reports on the neuroprotective effects of terrestrial polyphenols both in vitro and in vivo, studies exploring the neuroactivity of marine polyphenols are scarce. The great majority of the available works addressing the neuroactive properties of phlorotannins focus on the ones isolated from seaweeds of the genus *Ecklonia* (Laminariales), providing an exciting perspective for works to be developed with several other ecological and economically relevant species that remain unexplored.

Phlorotannins are particularly acclaimed as disease-modifying multifunctional agents that modulate the activity of CNS enzymes and neuronal receptors, also regulating signaling pathways linked to oxidative stress-mediated neuronal cell death and neuroinflammation. In fact, the outcomes from both in vitro and in vivo studies revisited in this review highlight that targeting multiple pathophysiological events may hold promise for future drug development, and phlorotannins are an auspicious basis to design new multitarget directed agents against neurological disorders. However, further in-depth studies are required, especially to ensure phlorotannin-crossing BBB permeability, a crucial factor in the development of CNS-active preparations.

In general, and despite several biotechnological challenges still ahead in order for phlorotannin-derived products to be effectively exploited as therapeutic and preventive agents, the high potentialities endowed to phlorotannins can be a starting point for neuro research ramp-up processes on these marine-derived products to address the challenge of neurodegenerative diseases.

## 4. Materials and Methods

In this review, we conducted a Scopus search to cover all the available studies, to present, on the experimental in vitro and in vivo neuroprotective effects of isolated phlorotannins and phlorotannin-rich extracts/fractions. The query terms used for the Scopus database search included the terms “phlorotannins”, “neuroprotection”, “neurodegeneration”, “neurotoxicity”, “cholinesterase”, “monoaminoxidase”, “tyrosinase”, “secretase”, “neuroinflammation”, “oxidative stress”, “memory”, “cognitive function”. Additionally, this search was complemented by further exploring the references of the articles retrieved from the Scopus search.
